# Tortuosity Index Based on Dynamic Mechanical Properties of Polyimide Foam for Aerospace Applications

**DOI:** 10.3390/ma12111851

**Published:** 2019-06-07

**Authors:** Sugeily Flores-Bonano, Juan Vargas-Martinez, Oscar Marcelo Suárez, Walter Silva-Araya

**Affiliations:** 1 Department of Mechanical Engineering, University of Puerto Rico-Mayagüez, Mayagüez, PR 00681, USA; sugeily.flores@upr.edu (S.F.-B.); juan.vargas5@upr.edu (J.V.-M.); 2 Department of Engineering Science and Materials, University of Puerto Rico-Mayagüez, Mayagüez, PR 00681, USA; 3 Department of Civil Engineering, University of Puerto Rico-Mayagüez, Mayagüez, PR 00681, USA; walter.silva2@upr.edu

**Keywords:** tortuosity index, polyimide foam, PolyuMAC^TM^, aerospace material

## Abstract

The determination of a reliable tortuosity index is lacking in the aerospace industry. Therefore, a methodology is formulated via direct and indirect characterization methods of a fluid-filled porous media. Chemical, thermal, and mechanical characterization was performed to the PolyuMAC^TM^ polyimide foam. Tortuosity was measured considering a pressure difference as the resistivity variable, rather than electrical resistivity or molecular diffusivity, as proposed on previous models. This is an empirical establishment of the tortuosity index considering the correlation among hydraulic and structural dimensionless parameters obtained through the Buckingham’s Pi theorem. The behavior of the polyimide was studied for samples of different lengths compressed at 30%, 60%, and 90% of its original length on the foaming direction. Results show that, porosity, sample length, and fluid viscosity are relevant for the insulation performance of the material. Regression analysis produced a significant statistical model fit to the data correlated from the dimensionless parameters for each dynamic compression series.

## 1. Introduction

The need of high temperature insulation polymers in the aerospace industry has led to the development of high-performance polyimide foam systems for various applications depending on its structure [1]. The PolyuMAC^TM^ polyimide foam insulation is a material developed in a joint effort between the NASA Langley Research Center and PolyuMAC TechnoCore, Inc. [2]. Its success is due to the flexibility in production and low manufacturing cost. The fabrication of this low-density and high-performance polyimide foam evolved into another generation of FPF-44 foam. This underwent ice mitigation tests on the insulation of liquid oxygen (LOX) feedline on the space shuttle external tank into the commercial production of the joint NASA LaRC-PolyuMAC™ foam [3]. This polyimide foam emerged from failed attempts to fabricate a composite material for a supersonic aircraft project [4]. They considered fabricating a new generation of insulation polyimide foams which resulted on the production of the TEEK (an acronym denoting the inventors’ names) technology. TEEK is a term used by NASA given to a polyimide insulator that can be found in powder form (precursor), friable balloon (intermediate material), and a foam [5]. First generation TEEK polyimide foams had a rigid structure manufactured using a friable balloon format, which allowed molding and curing it to the desired shape depending on the application. The chemical structure of this foam developed by NASA was improved into a new product FPF-44, also known as PolyuMAC^TM^. Unlike the former polyimide foam manufacturing technique, the new technology consists of a polyimide foam that rises at room temperature and cures via microwaving [4]. This TEEK technology derivation has acoustic and thermal insulation capabilities, self-extinguishing properties, and fire resistance and retardation. The foam is prepared from an aromatic of the polyimide precursor’s solid residuum and is composed by an expandable powder imidized upon the curing cycle [6]. This foam provided an effective insulation at cryogenic temperatures while maintaining a flexible structure. Moreover, this material is the first polyimide foam to rise at room temperature during the foaming phase. For the curing process, a mold with the desired shape (with the foam material) is exposed to microwave radiation of low intensity. This process reduced the manufacturing cost and increased the production rate [1,7].

Polymeric foams can be classified into two types, thermoplastic and thermoset. Polyimide foam is a highly cross-linked thermoset material. Their mechanical properties will depend on the glass transition temperature value of the polymeric material [8]. Knowing that polyimides have thermosetting matrices, one can expect a higher thermal resistance and higher thermal insulation [9]. Although, polyimide foams have been characterized in many ways, tortuosity is rarely mentioned. The concept of tortuosity can be simplified as the transport of a fluid through a complex configuration. This perspective allows a basic design of possible tortuosity models, depending on the investigation. C. Carman [10] introduced a tortuosity index intended to match experimental observations with a permeability model on granular beds influenced by the dimensionless analysis of parallel tubes [11]. For porous media, empirical tortuosity models have been proposed as a function of molecular diffusivity and electrical resistivity. For instance, an ideal tortuosity model for characterization of sandstone rocks was developed by Garrouch and Ali through diffusion and electrical measurements [12]. In the case of the polyimide foam, the acoustic insulation performance of the material on an aircraft double panel application was studied matching inverse characterization data with finite element predictions of transmission loss [2]. However, a model of tortuosity was not presented; instead, an optimization procedure on the finite element software COMET/Trim estimates the tortuosity.

Recent studies of foams for thermoacoustic applications demonstrated how tortuosity is very relevant to characterizing the material [13]. In this study, Napolitano et al. proposed an empirical approach. Additionally, this type of evaluation of acoustic properties is not restricted to polymeric foams. For instance, tortuosity is estimated through a diffusion technique applied to carbon foams [14]; in this case, the tortuosity value was assumed based on previous studies [15], but results in a methodology lacking practicality. Tortuosity becomes relevant in mass transfer applications due to the interaction of two mediums (gas/solid), and, by experimental measurements and numerical simulations, some authors proposed correlations for mass transfer in open-cell foams [16,17]. These types of materials (i.e., foams) possess great acoustic properties, that depend on their manufacturing methods and the characterization techniques used and that deal with the concept of tortuosity [18].

The present research focuses on the analysis of an open cell polyimide foam with flexible structure. If tortuosity can be defined as the ratio of the real-to-apparent distances that air flow has to travel through the foam, it can be correlated to sound absorption coefficients using microphones [19]. If the porous media (in our case the PolyuMAC foam) is intended for sound absorption applications (aircrafts, for instance), then the assessment of tortuosity in the porous media becomes very relevant. Since there is no standard method to measure tortuosity for porous media, we propose a methodology to define a tortuosity index. In addition, we provide a full characterization of PolyuMAC™ polyimide foam intended for aerospace applications. The material is evaluated as fluid is transported through the porous media.

## 2. Material Characterization

### 2.1. Sample Preparation

**Figure 1:**
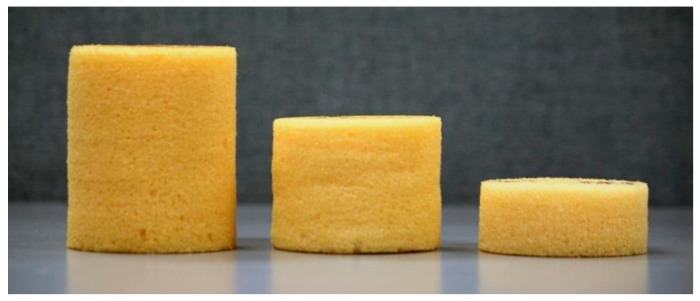
Foam cylinders after one, two, and three dynamic compressions.

PolyuMAC^TM^ foam panel was provided directly from NASA Langley Research Center in Hampton, VA. This foam has superb mechanical, acoustic, thermal, and flame-resistant properties. more detailed materials properties are available in the US patent “Polyimide foams” from Vazquez et al. [3]. The received 610 × 610 × 76 mm polyimide panels were cut into cylindrical shapes with a 63 mm diameter, as seen in Figure 1. In addition, these samples underwent one, two, and three dynamic compressions; these specimens are labeled in several graphics as PolyuMAC 1, 2, and 3, respectively.

### 2.2. Chemical and Thermal Characterization

Although polymers tend to be sensible to oxidation, depending on its chemical structure, this foam material can possess low sensitivity to oxidation [8]. As mechanical properties are influenced by the chemical structure, composition, and molecular configuration, chemical changes relate to oxidation and decomposition, while physical changes relate to glass transition and softening. Naturally, the polyimide composition has a relevant effect on its structural performance. Thus, the PolyuMAC™ thermal properties make it suitable for potential applications in the aerospace industry.

#### 2.2.1. Fourier Transform Infrared Spectroscopy

**Figure 2:**
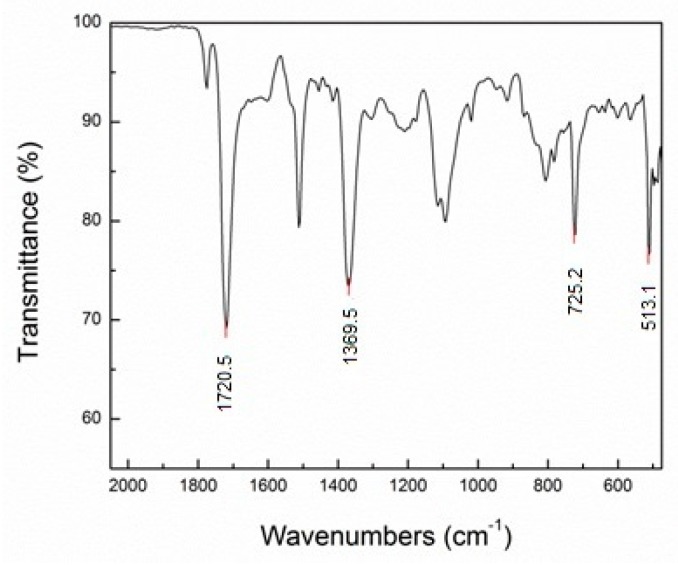
FTIR spectrum obtained from the polyimide foam.

Using the IRAffinity-1 spectrometer (Shimadzu, Kyoto, Japan), the spectra obtained was in the infrared radiation (IR) middle region of 4000–400 cm^−1^. The two main chemical bonds observed are: fingerprint on the 1500–600 cm^−1^ region, and double bonds on the 2000–1500 cm^−1^ region where carbonyl stretching can be recognized. Figure 2 displays the most intense peaks from the spectrum.

The FTIR spectra exhibit strong imide absorption bands at 1720, 1369, 725, and 513 cm^−1^. Table 1 summarizes the band assignment according to the region. The carboxyl bond represents a stretching vibration capacity of an aromatic with a symmetric imide wavelength at 1721 cm^−1^. Out-of-plane bending vibration of the imide ring is apparent as the sharp peak at 725 and 513 cm^−1^; at that range aromatic ring bends are expected. This spectrum is characteristic for highly aromatic polyimide foam and confirmed a thermoset material where mechanical compression produced no apparent effect on the polyimide structure.

#### 2.2.2. Thermogravimetric Analysis/Simultaneous Differential Thermal Analysis (TGA/SDTA)

**Figure 3:**
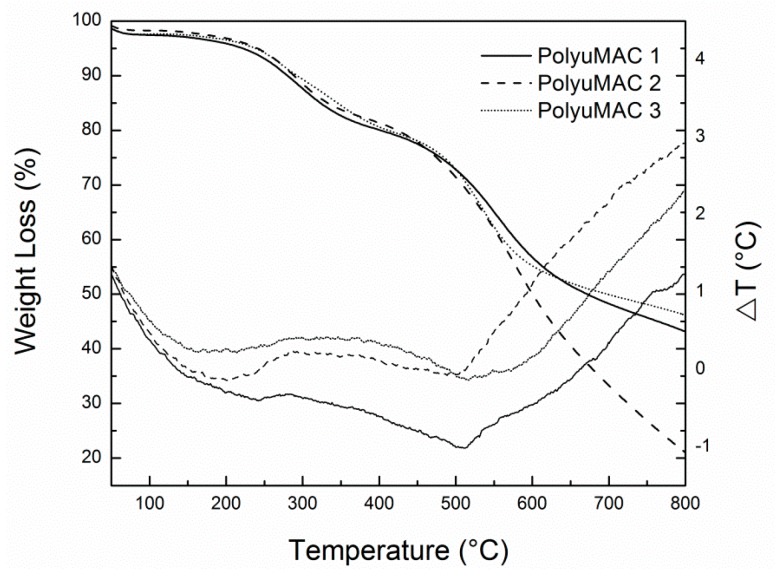
Thermogravimetric analysis (TGA) signal (left axis) and simultaneous differential thermal analysis (STDA) signal (right axis) of the polyamide samples.

The TGA/SDTA851e thermo-gravimetric analyzer (TGA, Mettler–Toledo, Columbus, OH, USA) allowed the thermal characterization of the PolyuMAC^TM^. Via the said TGA, we determined the thermal stability of the polymer by identifying at which temperature moisture evaporates from the sample without affecting the material’s structure. Three PolyuMAC™ samples of different weights (namely 7.42, 9.57, and 10.14 mg) were tested at 2.0 °C/min, with a lower limit of 26.59 ± 0.27 °C and an upper limit of 947.80 ± 0.27 °C. In addition, a simultaneous differential thermal analysis (SDTA) permitted to study the chemical, physical, and softening transition of the polyimide samples. Figure 3 shows the behavior of a thermoset polymer with curves representing typical multistage decomposition.

One must underscore, that in our TGA/SDTA instrument, the sensors, i.e., thermocouple and microscale, possess different sensitivity. Since the unit is mainly intended for thermal signals, the differential temperature readings bear more resolution. Yet, the plots allow verifying that smooth physical changes (weight changes) correlate with stronger thermal signals in the SDTA axis. This helps pinpoint events in the architecture of the foam. Further, according to Figure 3, weight loss due to moisture loss (nigh 100 °C) was around 3%. This information later became useful for the absorption test, where the sample must be dried without affecting the structure of the material. The decomposition temperatures for the various weights were 561.4 ± 34.4 °C. A significant endothermic peak around 500 °C on the SDTA plot suggests that the material started decomposition.

#### 2.2.3. Differential Scanning Calorimetry

**Figure 4:**
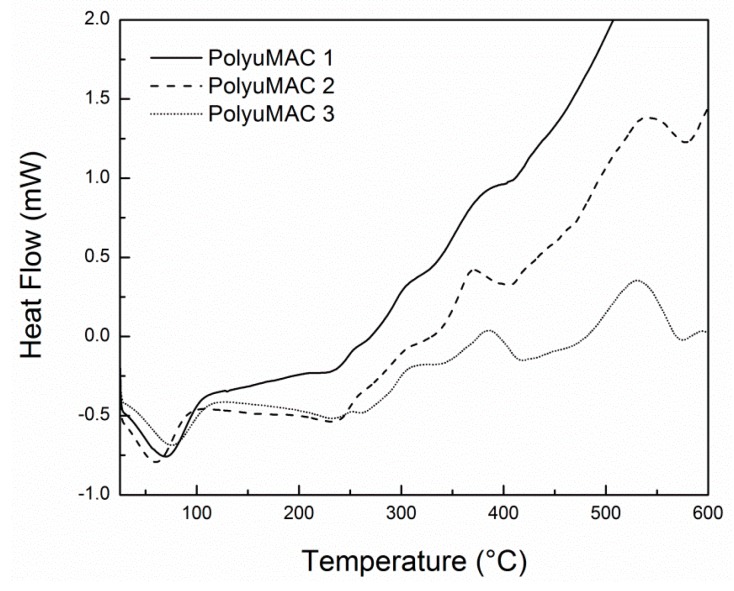
DSC results from the polyimide foam.

By assuming that the polyimide structure has an absorption or release capacity of heat, the glass transition temperature ($T_{g}$) can be obtained from the midpoint of the change in the slope of the baseline of the thermal signal. $T_{g}$ appears as a second-order endothermic step transition, instead of a peak. Above $T_{g}$, polymers have a higher heat capacity, which made it possible to measure the temperature range of this change which occurred at the glass transition. The ramp used in the differential scanning calorimetry (DSC, TA Instruments, New Castle, DE, USA) Q20 differential scanning calorimeter, furbished with an inert (nitrogen) atmosphere, was the same as for the TGA of 2.0 °C/min. The samples studied weighed 5.3 and 5.5 mg. As shown in Figure 4, the endothermic peak high of 100 °C confirms the moisture release observed on the TGA. Additionally, chemical oxidation is evidenced by the peaks at 350 and 500 °C. The softening temperature ($T_{soft}$) is reached on an endothermic transition around 400 °C, also identified on the TGA results.

### 2.3. Mechanical and Hydraulic Characterization

As mentioned, the many thermal and acoustic insulation properties of the PolyuMAC™ foam create a wider range of applications compared to common aromatic poroelastic materials such as commercial polyurethane and fiberglass. For these reasons, proper mechanical and hydraulic characterization is necessary to understand the interaction between the fluid and the solid phase. First, it is important to identify which factors affect each phase. The factors selected can be grouped as the structural form of the material (e.g., length, dynamic compressions, porosity, bulk density) and the macroscopic acoustic properties of the material (e.g., flow resistivity, fluid properties, tortuosity) [20].

#### 2.3.1. Absorption Test

**Figure 5:**
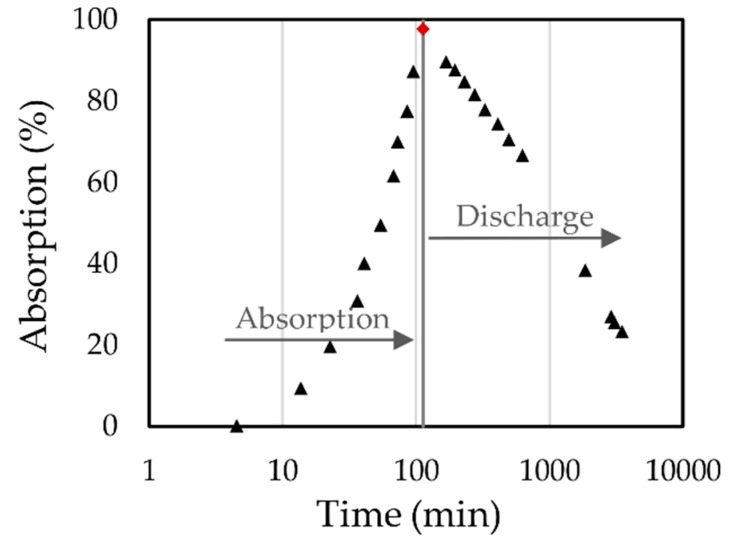
Absorption test results of polyimide foam.

The polyamide density was performed according to the ASTM D3574 [21] and its mean value was of 5.922 kg/m^3^ with a standard deviation of 0.325 kg/m^3^, and a median of 5.821 kg/m^3^. The experimental setup for absorption test was segmented into drying and saturation. Drying was performed in an oven for 10 min at 100 °C using the TGA results as reference. Then, the sample was transferred inside the glovebox where the humidity and temperature were constantly monitored to ensure a controlled system experiment. Inside the glovebox there was a precision balance and a spray nozzle with an attached syringe filled with methanol. This worked as a piston pump balancing the pressure inside the mechanism after the fluid was sprayed allowing collection of the exact volume being sprayed on the top of the sample. Water is commonly used for absorption tests. However, we opted to use methanol for its lower surface tension, which allows the alcohol to flow through the foam easier than water. Thus, the foam specimen was wet with a mist of methanol with a known volume to assess the absorption capacity of the material. One must underscore that the sample was dried prior to any test and, thereupon, saturated with methanol, which was confirmed when the foam started dripping (~113 min). After saturation, the sample was weighed to assess the fluid discharge. In addition, a control sample that was simply exposed to the environment was also placed inside the glovebox. No significant changes were observed in the control sample. The samples were weighed at each step of the process. The samples’ dimensions were 51 mm in length, 64 mm in diameter and had an average dry weight of 0.714 g according to the absorption test. The temperature inside the glovebox was 21.9 ± 0.14 °C and the humidity increased slowly with respect to time at a rate of ~0.033% min^−1^. Figure 5 shows an example of the behavior obtained from the sample.

#### 2.3.2. Dynamic Compressions.

**Figure 6:**
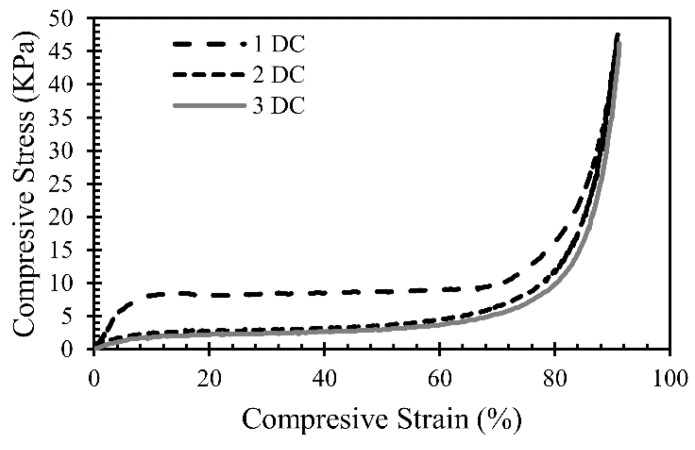
Stress vs. strain curve for foam samples with different dynamic compressions (DC), numbered as 1, 2 and 3.

The dynamic compression properties were determined according to ASTM D1621-10 [22]. An Instron^®^ 5944 allowed the compression tests to be carried out. As mentioned, the samples underwent compressions of 30%, 60%, and 90% of its original length on the foaming direction. At each percent, the samples were dynamically compressed (DC) through one, two, and three cycles. The deformation rate was 50 mm/min. The standard compression test suggests that the compressive strength is the stress at 10% deformation. This stress value represents the yield point and was identified on the plots acquired from this test. The Instron Bluehill^®^ software (version 3.25, 2010, Norwood, MA, USA) provides the plot of compressive stress and compressive strain (Figure 6). Young’s modulus seen in Table 2 is observed on the linear elastic region of the plot. The slope is measured as the ratio between change in compressive stress and strain. As expected, since the compression percent of the foam raised their densification, the mechanical strength of the material also rose [23].

**Figure 7:**
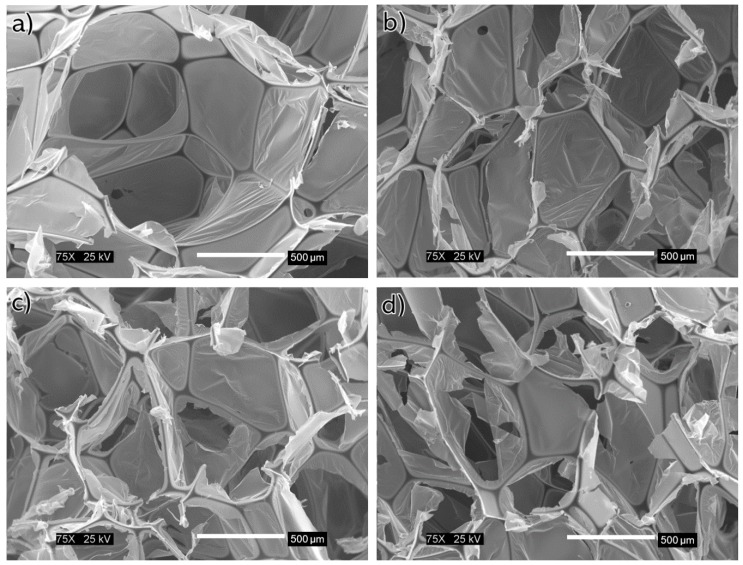
Scanning electron microscopy (SEM) images of foam samples: (**a**) as provided, (**b**) after one dynamic compression to 30% of its length, (**c**) after two dynamic compressions to 30% of the original length, and (**d**) after three dynamic compressions to 30% of its length.

The thermoset flexible material was expected to recover its length after 24 h to then undergo the flow resistivity test. A preliminary examination showed that for most samples the recovery was 95%. Scanning electron microscopy (SEM, JEOL-7000, Peabody, MA, USA) micrographs were used to observe the effect of the dynamic compressions on the structure. Figure 7 allows comparison of control sample against the dynamically compressed ones.

#### 2.3.3. Porosity

**Figure 8:**
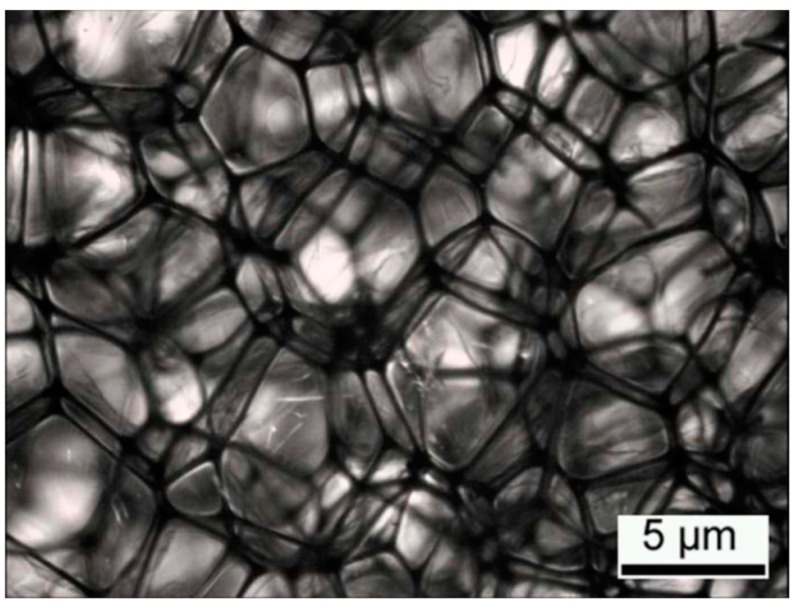
Optical stereoscope image, 25 mm thick sample with 30% and 2 DC.

Quantitative image analysis allowed the porosity to be measured (Figure 8), which normally has a value of 99% for acoustic insulators [2]. Micrographs of the samples were obtained using a SMZ1500 stereomicroscope (Nikon, Melville, NY, USA) with diascopic stand, furbished with a shadow free Fiber-Lite MI-150 top illumination, and a V-Lux compact cold light source to complement the Fiber-lite. Afterwards, the porosity was determined using the ImageJ software. Porosity (ϕ) is defined as a volume fraction, according to Equation (1):

(1) $$\phi =1-\frac{V_{F}}{V_{S}}$$

where $V_{F}$ the is the volume of the foam and $V_{S}$ is the bulk volume of the sample. The porosity area $A_{p}$ was determined from the image analysis as well as the total area of the image $A_{T}$. The porosity for the PolyuMAC™ foam was 0.985.

#### 2.3.4. Flow Resistivity

**Figure 9:**
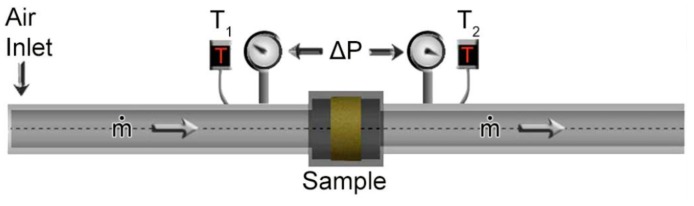
Flow resistivity test setup.

Flow resistivity was determined according to ASTM C522 [24]; a constant 55 kPa pressure was controlled from the air compressor allowing a flow rate of approximately 0.31 m^3^/min, a value selected after performing a pilot test. The pressure drop was measured from two manometers located at each end of the sample length (Figure 9). This test also allowed determining the air bulk density ($\rho_{air}$) and the permeability (κ). The structure of the foam creates resistance in the fluid flow as it raises the pressure drop across the specimen. Said resistance is a function of the foam specimen size, deformation percent, and number of dynamic compressions. The direct effect of the number of compressions per compression percent is difficult to analyze because of the material’s recovery capacity after deformation. The results from the flow resistivity test were analyzed by variation of length and variation of dynamic compressions (DC). Table 3 presents the pressure drop measured in the test.

According to Darcy’s law, the pressure drop can be defined as $\Delta P=r_{f}\;V\;L$, where $r_{f}$ represents the flow resistivity, V the air velocity through the porous medium, and L the sample length [25]. Equation (2) permitted $r_{f}$ to be determined, where some variables are changed.

(2) $$r_{f}=\frac{\Delta P\;\rho_{\mathrm{air}}\;A}{l\;\dot{m}}.$$

The modified Darcy’s law can be written as Equation (3).

(3) $$Q=-\frac{\kappa \;A}{\mu \;l}\Delta P.$$

**Figure 10:**
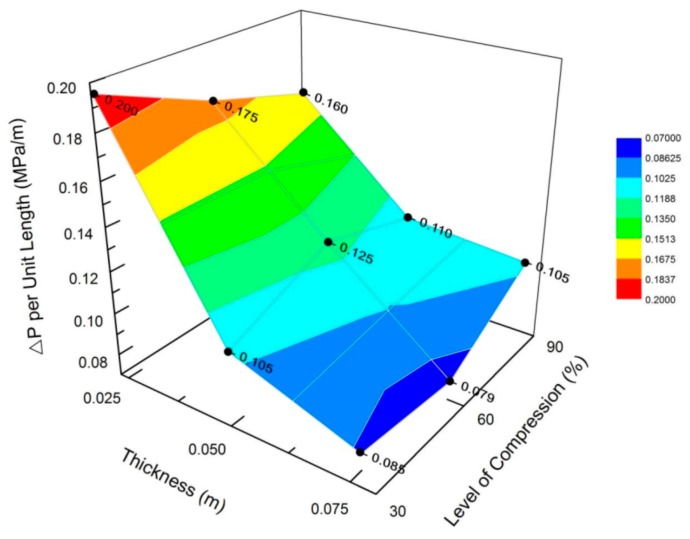
Pressure drop as a function of the sample thickness and the compression percent.

Figure 10 illustrates the behavior pressure drop per unit length presented for each percent of compression. One must expect that the pressure drop be proportional to the sample length.

### 2.4. Determination of Tortuosity Index

Tortuosity represents the energy absorption capacity of a porous media; such capacity could be either thermal or acoustic. Several models have been proposed considering the porosity as a relevant factor that affects tortuosity [12,26]. Conventional poroelastic models, including transmission loss, have been used to predict the acoustic insulation performance of the polyimide foam. Further, tortuosity can be evaluated as the ratio of a fluid passing through the structure’s interconnectivity and the same fluid passing without the porous media. The complexity of the path traced by the fluid through the structure is also known as tortuous path. Hence, porosity is an intrinsic property of the polyimide foam structure. An alternative characterization method must be developed to measure tortuosity since there is no standard method.

Furthermore, the mechanics of fluid-filled porous media can be described by the Biot theory of poroelasticity, which analyzes the diffusion-deformation by the influence of a fluid and a solid phase. Therefore, a conceptual model is attainable for a known fluid moving freely through the interconnectivity of a porous material. Biot parameters represent the macroscopic properties that describe fluid filled porous media interactions. The implementation of Buckingham’s Pi theorem allows simplifying a complex porous system by reducing the conceptual model to independent components that are described by the Biot parameters.

#### 2.4.1. Estimation of Pi Terms

**Figure 11:**
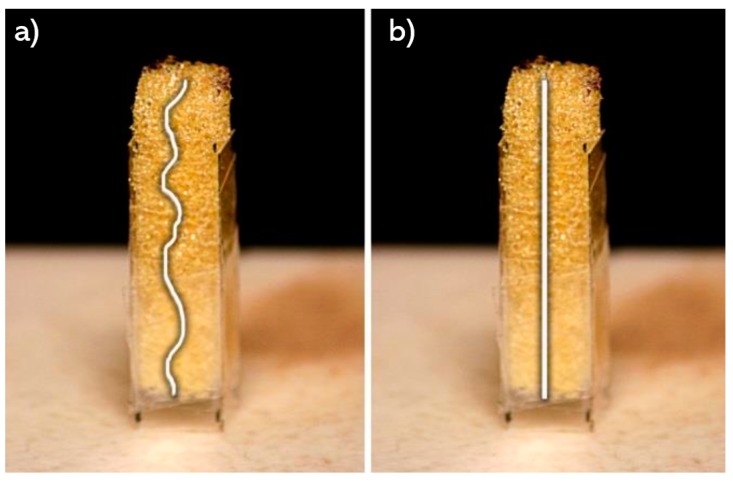
(**a**) Length of path ($l_{P}$) traced by the fluid through porous media; (**b**) length of path (l) by the fluid without porous media (sample length).

Fluid/solid interaction can be simplified as the ratio of path length of fluid ($l_{P}$) through the porous media and the sample length (l), as shown in Figure 11. Therefore, a higher tortuosity index justifies a more complex path for the fluid, which one interprets as better insulation capacity. The tortuosity index cannot be equal to one because such occurrence would mean that a material lacks a tortuous path. The interaction between these two continuums can be explained through the Pi theorem via experimentation to calculate a tortuosity response. An analysis of variance (ANOVA) and regression permitted to identify regression equations and determine which one possessed the most suitable (largest) coefficient of correlation [27].

Through the Pi theorem we can express n dimensional variables as n−k dimensionless variables. The dimensional variables of interest in our case are fluid density, foam density, dynamic viscosity, velocity, sample diameter, pressure change, sample length, and length of path ($\rho_{l},\;\rho_{f},\;\mu_{l},\;V,\;D,\;\Delta P,\;l$, and $l_{P}$ respectively). They can be expressed in k=3 reference dimensions, mass (M), length (L), and time (T). Hence, there are five Pi terms, namely $\pi_{i}$ with i=1 to 5 since $l,\;\rho_{l}$, and V contain reference dimensions, and they were selected as repeated variables. Then, applying the Buckingham Pi theorem the terms become those presented in Equations (4)–(8).

(4) $$\pi_{1}=\frac{l_{P}}{l},$$

(5) $$\pi_{2}=\frac{\rho_{f}}{\rho_{l}},$$

(6) $$\pi_{3}=\frac{\Delta P}{\rho_{l}\;V^{2}},$$

(7) $$\pi_{4}=\frac{D}{l},$$

(8) $$\pi_{5}=\frac{\mu_{l}}{l\;\rho_{l}\;V}.$$

These Pi terms represent tortuosity, specific gravity, pressure coefficient, shape factor of sample, and the inverse of Reynolds number, respectively from $\pi_{1}$ to $\pi_{5}$. The main purpose is to express the tortuosity as a function of the other π terms. The specific gravity is a material property; therefore, in our case this term is a constant. The estimation of tortuosity index cannot be determined from Equation (4), since $l_{P}$ cannot be measured from direct methods. Assuming the possibility of observing bulk properties produced by microscale properties, the parameter of tortuosity can be measured indirectly. The formulation of an empirical model for fluid-filled porous media is possible using a known fluid moving freely through the interconnected pores. Due to the impossibility to measure $l_{P}$ directly, we can use Pirson’s model to determine the tortuosity [28]. Then, the $\pi_{1}$ term becomes Equation (9).

(9) $$\pi_{1}\;=\;\tau =\sqrt{\frac{r_{f}}{r_{a}}\;\phi }.$$

In Equation (9), $r_{f}$ represents the flow resistivity with the polyimide foam inserted and $r_{a}$ the flow resistance of the viscous fluid without the obstruction. These parameters were previously measured in the experiment in Figure 9, whereas the material porosity was computed through image analysis from the stereomicroscope photographs. The results of tortuosity are summarized in Table 4 and the pressure coefficient in Table 5.

**Figure 12:**
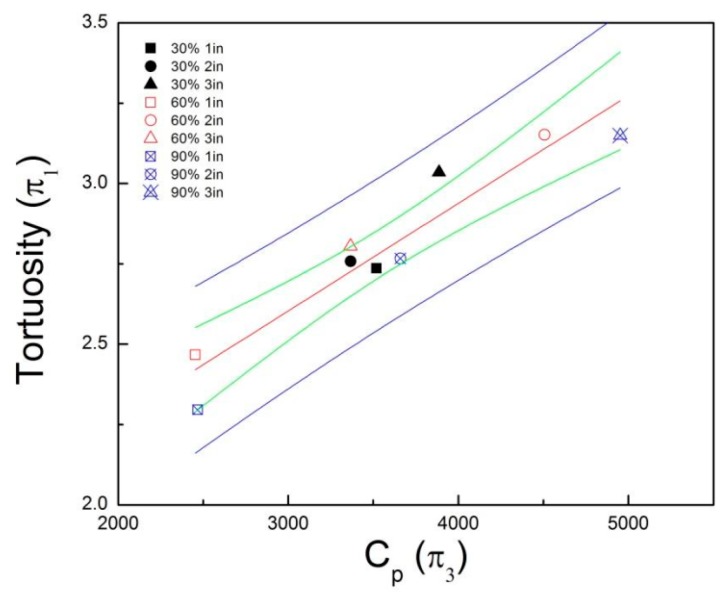
Confidence intervals for the correlation between $\pi_{1}$ and $\pi_{3}$.

The results depict a linear dependence between the pressure coefficient and tortuosity. A higher pressure drop means that the fluid went through the sample via a longer path. Hence, more inertia entails a higher tortuosity. In Figure 12, confidence intervals of 95% are presented with the green constraint for the mean value of the linear fit (red line) to demonstrate an estimated range of uncertainty related to the experimental data collected. All the values of a 95% prediction interval for a single value of $\pi_{1}$ is represented between the blue bands. The R^2^ values for the $\pi_{1}$ and $\pi_{3}$ parameters’ relation was 0.759 for 30% compression, 0.993, for 60% compression, and 0.986 for 90% compression.

When the polyimide is compressed, the pore walls are brought together but this did not appear to affect the overall porosity after complete or partial recovery. On the other hand, the tortuosity increased along with the dynamic compressions because the length decreased. For this geometric factor the flow must pass through a reduced area of high porosity. Consequently, there is an inverse relationship between the tortuosity and the sample length. The term $\pi_{5}$ is focused on the fluid phase, as constant air flow, ideally, passed through all available space on the porous media structure. However, the SEM micrographs (Figure 7) revealed a thin membrane that might affect the path of such air flow. This suggests that these thin membranes (unaffected after the dynamic compressions) are on the fluid’s direct paths. Therefore, the airflow can continue through the path by breaking the membrane or can diverge to another path following the pore walls. Comparing with previous models [12], the molecular diffusivity is likely to experience the same conditions when a gas is not always carried through the most direct path.

#### 2.4.2. Data Regression Analysis

Multiple linear regression analysis was used to assess the possible association between the regressors and the dependent variable. In this research, $\pi_{1}$ (i.e., the tortuosity index) was measured experimentally with the air flow resistivity setup. The variation of dynamic compressions, percent of compression deformation, and length represent a particular case, which is related using the function obtained from Buckingham’s Pi theorem where $\pi_{1}=f\left(\pi_{3},\pi_{4},\pi_{5}\right)$. Equation (10) presents this general relationship.

(10) $$\pi_{1}=b_{0}+b_{1}\pi_{3}+b_{2}\pi_{4}+b_{3}\pi_{5}+l+\eta ,$$

where $b_{i}$ represents the regression coefficients; l, the sample length; and η is the percent of compression deformation (with respect to its original length). A best subset regression strategy for each DC allowed observing the single and interaction effects. Thus, different models were identified by varying terms and comparing the statistical significance described by R^2^, R^2^_adj_, and the standard deviation (S). Table 6 presents the best subset regression for the samples with 1 DC.

A better model fit is achieved when the three variables are considered. $C_{P}$ ($\pi_{3}$) explains ~88% of the tortuosity in this model, and a model with $C_{P}$ and Re^−1^ ($\pi_{5}$) explains almost 92% of the response, since shape factor ($\pi_{4}$) has a low contribution. Table 7 and Table 8 show the best subsets for 2 and 3 DC.

The contribution of $\pi_{3}$ becomes more significant when the number of DC increases. Therefore, we can create a model for 1 and 2 DC using $\pi_{3}$ and $\pi_{5}$, and a model for 3 DC using only $\pi_{3}$. Table 9 shows these models to explain the tortuosity index of the foam, and Table 10 shows the regression equations.

## 3. Discussion

The characterization methods employed corroborated the chemical and thermal properties of the PolyuMAC^TM^ polyimide foam. In effect, this material possesses a high heat resistance even at temperatures higher than 500 °C, which gives it a wide range of potential applications. The FTIR spectra displayed a characteristic result for aromatic polyimides with high $T_{g}$. The thermal stability and resistance to oxidation degradation were successfully validated by a $T_{g}$ of 294.2 ± 9.9 °C. As aforementioned, the new TEEK technology allows producing light polyimide foams with a density ranging between 3.2 and 16 kg/m^3^ [2]. With a density of 5.922 kg/m^3^, the material under study falls within such range with a standard deviation of 0.325 kg/m^3^. At this point, one should note that density is not a measure of the foam structure rigidity. Instead, density is one factor reflecting the quality and performance of the material. For instance, in a study of polyimide foam used as a double panel for sidewall applications by Silcox et al. [2], a sample with a density of 6.4 kg/m^3^ compressed at different percentages showed an average Young’s modulus of approximately 75, 95, and 130 kPa for 30%, 60%, and 90% compression levels, respectively. These values are comparable with the outcomes of the present research, where the mean Young’s moduli were 78, 93, and 180 kPa for 30%, 60%, and 90% compression levels, respectively. In that same study of double panels at the Gulfstream Acoustic Test Facility, a 9.6 kg/m^3^ density sample compressed at 10% of its original length had a tortuosity of 3.11 and for samples of 5.4 kg/m^3^ compressed at 20% of its original length presented a tortuosity of 1.02. The samples in this study, compressed at 30% from its original length had an approximate tortuosity of 3.05. The method to determine the tortuosity is not specified by Silcox et al., but we can infer that density and compression level affected such tortuosity.

At this point, one must acknowledge that a more extensive assessment of the polyimide oxidation sensitivity would have been suitable. Unfortunately, that experimentation would need a thermogravimetric analyzer operated with air atmosphere and interfaced with a Fourier transform infrared spectrophotometer for the simultaneous characterization of evolved volatiles [29]. Our TGA unit lacks such capability and only produces a differential weight or weight loss versus temperature curve.

In our research, our empirical model used the pressure difference as an analogous approach for a resistivity factor. In effect, one can observe in the surface plot of Figure 10 that the pressure drop changes with the sample length and compression percent. According to Darcy’s Law, the parameters involving pressure drop can describe the effect of the fluid velocity passing through the interconnected open cells. A lower value of permeability can then be considered as a lower flow rate of the fluid phase through the solid phase. The tortuosity barely fluctuates between dynamic compressions because of the almost constant porosity parameter, as explained before. On the other hand, the permeability increased with the sample length. Hence, the slope obtained in the linear fit per unit length resulted in an inverse relationship between κ and τ. The slope is affected by the permeability. Samples with 76 mm (3 in.) length had more volume than samples with 25 mm (1 in.) length; hence, the pressure drop was higher in the 76 mm samples, justifying the hypothesis of a velocity attenuation as the fluid flows through the porous media at a constant rate.

A regression model for the dynamic compressions was obtained using an analysis of variance. The residuals of this lineal regression met the assumptions of normality, equal variances, and independence. Therefore, a regression equation was produced for the estimation of tortuosity index at different dynamic compressions. The R^2^_adj_ of the regression equation increased and the standard deviation decreased with the number of dynamic compressions. These results indicate that the 3 DC model was statistically more significant than those obtained for less dynamic compressions. Still, further research is required to thoroughly study the tendency for more than 3 DC. These models were simplified by reducing the number of regressor variables without diverting from the original purpose of the analysis using a best subsets regression strategy. Since the technique selects the best combination of regressor variables at different levels, reasonable and simpler models are developed so as to explain more effectively the tortuosity at different dynamic compressions.

The values of R^2^ and R^2^_adj_, as well as the standard deviation were the principal criteria for the selection of the variable combination. The two variables that explained the variability of tortuosity the most they were $\pi_{3}$ and $\pi_{5}$. For 1 DC and 2 DC, the R^2^_adj_ for the new models were 91.3% and 94.3%, respectively. However, the model for 3 DC had a better correlation with an R^2^_adj_ of 95.7%. In addition, these two variables were able to explain the interaction between the fluid and the solid phase. The reason why $\pi_{4}$ was eliminated by the best subsets’ regression is because the parameter is a geometry factor of the sample. Intrinsically each sample carries the effect after deformation at a compression percent when it is characterized on the flow resistivity test.

The method presented in this study considers the pressure coefficient as well as the Reynolds number as the necessary factors to determine the tortuosity index. This low-cost procedure allowed establishing that other factors do not have enough relevance in determining the tortuosity of the PolyuMAC™ foam. Thus, by an indirect yet economical means, the tortuosity index can be determined via the flow through the porous media. This presents an advantageous alternative to test this material, as flow properties can be employed to indirectly obtain the foam tortuosity. In addition, we think that this method can be readily applied to other foam materials because the factors used to determine the tortuosity index were independent of the foam nature, as long as sample is highly porous.

## 4. Concluding Remarks

This work presents a multifarious assessment of the PolyuMAC^TM^ polyimide foam used for insulation and acoustic absorption in aerospace components. The material was provided by NASA Langley Research Center “as is,” i.e., without prior compressions.

Dynamic compressions of 30%, 60%, and 90% of the foam’s original length allowed the mechanical response of the foam to be evaluated. As the compression percent of the specimens increased, so did their densification. This led to higher strength on the more deformed specimens. Although this flexible material was expected to recover its full length after 24 h, most samples recovered 95% of their initial length. Scanning electron microscopy confirmed the effect of the dynamic compressions on the structure and the damage of some ligaments.

Chemical and thermal characterization of the PolyuMAC^TM^ foam demonstrate the importance of this material type as an insulator, since it has softening and decomposition temperatures over 400 and 560 °C respectively. In addition, this foam possesses a very low density, causing also a high absorption capacity (of a low surface tension fluid) of over 97%. As an aromatic polyimide, the foam is apt for flexible structures that withstand high temperatures without losing fire retardation capacity. As expected, the collected FTIR spectra were characteristic of a highly aromatic polyimide foam and confirmed a thermoset material where mechanical compression produced no apparent effect on the polyimide structure (architecture).

Tortuosity, a complex parameter that describes the energy absorption capacity of the material, cannot be measured directly. Therefore, the present research proposes an alternative methodology for the determination of the tortuosity index via indirect characterization of the porous structure of a PolyuMAC^TM^ polyimide foam. This empirical model depends only on the pressure coefficient and Reynolds number of a fluid passing through the open cell structure of the foam. The methodology based on an empirical model uses the Biot parameters describing the interaction between a fluid and a solid phase for flow transport through porous media. Quantitative image analysis permitted to measure the porosity of the open cell structure of the foam. Straightforward analysis methods, such as Biot poroelasticity model and Darcy’s law, allowed registering macroscopic parameters defined on Pi parameters. An analysis of variance was the statistical analysis used to correlate the Pi terms. The factors represented by (a) the number of dynamic compressions and (b) the length of the samples required an independent evaluation for better understanding. The recovery capacity of the material was a component to help explain the lack of variance between the dynamic compressions.

## Figures and Tables

**Table 1:** Important frequencies of IR absorption.

Frequency (cm^−1^)	Bond Type	Vibration of Imide Ring
1721	C = 0	Symmetric stretching
1369	C – N	Stretching
725	C = 0	Bending
513	C – C	Bending

**Table 2:** Mechanical properties of the samples compressed at various percentages of their original length.

Compression Percent (%)	E	σ at 10% (KPa)
30	78	5.5
60	93	5.7
90	180	8.7

**Table 3:** Observations of pressure drop results in Pa by the flow resistivity test.

DC (%)	Height (in)	Pressure Drop (Pa)		
		1 DC	2 DC	3 DC
30	1	5285.98	4021.94	5745.63
	2	5285.98	5228.52	5515.81
	3	6492.56	6722.39	6435.11
60	1	4251.77	4763.13	4481.59
	2	6952.21	5056.16	6894.76
	3	5515.81	6664.19	6090.37
90	1	3562.29	2987.73	4826.33
	2	5285.98	6320.19	4596.50
	3	7584.23	8273.71	8273.71

**Table 4:** Results of tortuosity at different dynamic compressions.

DC (%)	Height (in)	Tortuosity		
		1 DC	2 DC	3 DC
30	1	2.70266	2.36944	2.80217
	2	2.72677	2.68492	2.78236
	3	3.01624	3.06692	3.00523
60	1	2.44295	2.54861	2.48919
	2	3.12294	2.66704	3.09503
	3	2.78336	3.05896	2.92725
90	1	2.22186	2.03276	2.60496
	2	2.71777	2.98123	2.51618
	3	3.25924	3.18329	3.41140

**Table 5:** Pressure coefficient ($\pi_{3}$) at different dynamic compressions.

DC (%)	Height (in)	Pressure Coefficient		
		1 DC	2 DC	3 DC
30	1	3414.56	2365.36	3411.50
	2	3267.2	3024.92	3407.58
	3	3770.5	4030.00	3881.04
60	1	2380.74	2695.76	2436.16
	2	4372.42	3065.58	4430.74
	3	3265.5	4022.46	3746.52
90	1	2168.46	1989.792	2854.08
	2	3460.54	3991.16	2996.40
	3	4733.48	4744.44	5364.30

**Table 6:** Best subsets regression for 1 DC.

Model ID	N. of Terms	R^2^	R^2^_adj_	S	$\pi_{3}$	$\pi_{4}$	$\pi_{5}$
A	1	88.8	88.3	0.125	X		
B	1	36.4	33.9	0.298		X	
C	2	91.9	91.3	0.108	X		X
D	2	91.4	90.7	0.112	X	X	
E	3	9.2	94.6	0.085	X	X	X

**Table 7:** Best subsets regression for 2 DC.

Model ID	N. of Terms	R^2^	R^2^_adj_	S	$\pi_{3}$	$\pi_{4}$	$\pi_{5}$
A	1	93.2	92.9	0.123	X		
B	1	54.3	52.5	0.318		X	
C	2	94.7	94.3	0.110	X		X
D	2	94.4	93.9	0.114	X	X	
E	3	97.8	97.5	0.0736	X	X	X

**Table 8:** Best subsets regression for 3 DC.

Model ID	N. of Terms	R^2^	R^2^_adj_	S	$\pi_{3}$	$\pi_{4}$	$\pi_{5}$
A	1	95.9	95.7	0.082	X		
B	1	14.6	11.0	0.375		X	
C	2	96.2	95.9	0.081	X		X
D	2	96.1	95.8	0.081	X	X	
E	3	97.9	97.6	0.061	X	X	X

**Table 9:** Comparison of statistical significance for models with one variable ($\pi_{3}$) and two variables ($\pi_{3}$ and $\pi_{5}$).

	$\pi_{3}$			$\pi_{3}$ and $\pi_{5}$		
DC	R^2^	R^2^_adj_	S	R^2^	R^2^_adj_	S
1	88.8	88.3	0.125	91.9	91.3	0.108
2	93.2	92.9	0.123	94.7	94.3	0.110
3	95.9	95.7	0.082	–	–	–

**Table 10:** Simple models identified by the regression analysis.

DC	Regression Equation	
1	τ=1.95+(5.58×10−4) CP−653/Re	(11)
2	τ=1.82+(6.38×10−4) CP−708/Re	(12)
3	$\begin{array}{c} \tau =1.63+\left(6.71\times 10^{-4}\right)\;C_{P} \end{array}$	(13)
